# Evaluation of the Perceived Benefits of a Peer Support Group for People with Mental Health Problems

**DOI:** 10.3390/nursrep14030124

**Published:** 2024-07-12

**Authors:** David Beard, Charlie Cottam, Jon Painter

**Affiliations:** Department of Nursing and Midwifery, Sheffield Hallam University, Sheffield S10 2BP, UK; c.cottam@shu.ac.uk (C.C.); j.painter@shu.ac.uk (J.P.)

**Keywords:** peer support, depression, service evaluation, group

## Abstract

This paper reports on a service evaluation of PeerTalk, a nationwide charity that organises and facilitates peer support groups for individuals with depression. Therefore, the aim was to gather and synthesise benefits perceived by support group attendees. Thematic analysis was undertaken following the collection of data from two group interviews comprising PeerTalk support group attendees. Once those data were analysed, five key themes emerged: (1) talking/listening, (2) socialising, (3) contrast with other services, (4) personal benefits, and (5) structure and accessibility. Two further minor themes were also identified: (6) wider benefits and (7) areas for development that could lead to overall improvements to the service. PeerTalk’s support groups provide multiple opportunities for attendees to meet others who have similar experiences within an environment that does not require formal engagement or commitment. Those that attend find benefit from supporting others and socialising within the group. These benefits are complementary to mainstream services that they may concurrently be involved with, rather than replacing or hindering them. Peer support groups can therefore provide a resource for healthcare professionals to which they can direct individuals who may feel benefit from engaging with other individuals with similar experiences. Sheffield Hallam University granted ethics approval for the study (ER:59716880) prior to its commencement (16 February 2024).

## 1. Introduction

Depression can vary in severity, but is always typified by a negative impact on thoughts, feelings, actions, and perceptions of the world. A clinical diagnosis is made on the basis of symptoms such as sadness, irritability, feelings of helplessness, worthlessness, hopelessness, and anhedonia, sleep and appetite change, and reduced energy and concentration levels [1].

Major depression is widespread, estimated to impact approximately 3.8% of the global population, which equates to approximately 280 million individuals [2]. Women bear a disproportionate burden of depression compared to men, with maternal depression in developing countries hindering child growth [3], reverberating across generations and impacting national growth [4]. Preventative measures like education, problem-solving, social support, and reminiscence interventions can mitigate risk factors [5,6,7]; however, the condition is likely to prevail.

Various explanatory models of depression exist within the biopsychosocial framework, each supported by empirical evidence. This multidimensional model posits that psychological disorders arise from interactions between genetic, biological, psychological, and environmental factors [8]. Davidson et al. [9] propose a tripartite model integrating individual, familial, and socio-cultural factors to understand depression, emphasising the complex interplay of biological, psychological, and social elements. Considering each element in turn; neurochemical imbalances, particularly involving serotonin, noradrenaline, and dopamine, are implicated in depression [10]. Biological research has long focused on neurochemical abnormalities and changes in hormonal secretion patterns [11,12]. Evolutionary system theory suggests that depression is an adaptive response to social risks and insecurities [13,14,15]. Psychologically, depressive symptoms can significantly impact personality and lead to serious consequences, especially during adolescence, a pivotal developmental period [16]. Mental disorders, prevalent among young people, underscore the need for greater attention to adolescent mental health [8]. The transition from childhood to adulthood involves complex social and psychological challenges, contributing to vulnerability and potential psychological disorders [17]. Finally, decreases in social support can compromise an individual’s defence system, contributing to feelings of helplessness and isolation, potentially impacting health [18]. Social support networks play a crucial role in mitigating the impact of stressful events and reducing the risk of depression and anxiety [19].

While antidepressants are effective in treating depression, many individuals do not achieve full remission, necessitating additional/alternative treatments [20]. Research into effective, cost-efficient alternatives and/or augmenting treatments for depression is therefore crucial to improve the health and well-being of millions [9]. Social networks, defined by Lloyd-Evans et al. [6], offer instrumental and emotional support to individuals comprising various individuals, functions, and situations tailored to different needs. As a consequence, they provide a potentially important means to ameliorate the impact of depression.

Social support encompasses resources provided by others during times of need and is studied across disciplines that embody the biopsychosocial model, i.e., medicine, psychology, and sociology. Initially recognised in the 1970s, the association between social ties and health highlights the importance of support from various groups, such as family, friends, and neighbours, offering affection, assistance, and information to foster feelings of love, esteem, and security [18]. Against a backdrop of increased demand for mental health services [21] and in keeping with the notion of empowerment of people with mental health conditions [22], peer support has emerged in the UK (and elsewhere) as a way to augment and in some instances even replace traditional service provision.

Peer support services can have varying durations, locations, and content, and involve selected voluntary or professional peers with effective communication skills, disease understanding, and compassion [23,24]. Research indicates that such services can improve social function, quality of life, and self-efficacy in individuals with severe mental illness [25], with peer education enhancing patient compliance and self-awareness in depression treatments specifically [26,27]. Peer support can also reduce hospitalisation rates and recurrence of depression compared to traditional methods [28,29]. Additionally, it fosters mutual understanding, empowering patients through shared experiences and successful problem-solving [6,30]. Peer support mitigates loneliness, enhances self-care and empowerment, and operates through mechanisms such as reducing isolation, buffering stress, and providing positive models [23]. Despite these benefits, barriers to access and integration into mainstream treatment pathways persist, with a lack of systematic evidence hindering wider adoption [6].

PeerTalk is a national charity that offers peer support groups for individuals dealing with depression and related mental health issues. Inspired by the Irish charity Aware, PeerTalk was founded in 2014 with the goal of establishing a sustainable network of peer support groups throughout England to directly enhance the well-being of attendees and indirectly combat the stigma surrounding mental illnesses. PeerTalk also aims to promote positive narratives about mental health through its support groups, raising awareness and challenging stigma by empowering peers to share their stories, learn from one another, and offer each other support. Its first support groups launched in Bradford and Preston in 2016. Their groups operate on the principle that peer relationships, rooted in shared experiences, create a unique environment for recovery and foster optimism and hope. Typically meeting once a week, groups are facilitated by two trained volunteer facilitators who prioritise the creation of a safe space for attendees to share their experiences for mutual support, without providing counselling or advice themselves.

The charity has an online presence; however, healthcare professionals such as mental health nurses can also direct individuals to these services during interactions with the people referred into their mainstream services. PeerTalk can then become part of an overall therapeutic response with regular access that does not require a formal referral or have a significant waiting time. Given the national variability in peer support services, their integration into tradition mental healthcare, and the relative paucity of definitive empirical evidence, PeerTalk sought to better understand the impact of its provision by building on the results of a previous survey of attendees. It therefore commissioned Sheffield Hallam University to conduct an independent service evaluation to gather and synthesise responses from those attending their support group meetings regarding the benefits they experienced. This report outlines the process, findings, and potential implications of this evaluation project.

## 2. Materials and Methods

### 2.1. Study Design

To gain the perspectives of PeerTalk’s support group participants, a qualitative methodology was employed. Qualitative research is rooted in naturalistic exploration, delving into individuals’ experiences through the use of words and text for data collection and analysis [31]. As the project aimed to gauge the impact of PeerTalk’s support groups, a service evaluation approach, endorsed by the World Health Organization [32], was adopted. This method entails a systematic and unbiased assessment of the organisation’s achievements, practices, and contextual factors to illuminate areas of success or potential improvement. The evaluation sought to provide evidence-based, credible, and reliable findings to allow PeerTalk to enhance service quality and hence its user experiences. To achieve this, the overarching design of the cross-sectional service evaluation was that of joint/group interviews, an increasingly common method in healthcare research (Szulc and King 2022 [33]; Green and Thorogood 2018 [34]), wherein two (dyads) or three (triads) of participants engage in small-group discussions and share personal insights, facilitated by a moderator to maintain focus and ensure all perspectives are captured (Morgan et al., 2016 [35]; Szulc and King, 2022 [33]; Finch et al., 2023 [36]).

### 2.2. Recruitment and Sampling

Recruitment was undertaken by a process of convenience sampling, with PeerTalk advertising the group interviews by sending out an invitation to all of its members who attend these support groups in two localities. The two localities were selected due to specific requests from the commissioners regarding these two support groups. Their aim was to understand more about their impact specifically. This allowed attendees to decide whether to engage or not. All participants were required to be over the age of eighteen, to have regularly attended a PeerTalk-facilitated group, and to have access to the internet so that they could participate in the online data collection.

### 2.3. Data Collection

As the charity had previously undertaken a survey of its attendees, a more in-depth method of data collection was deemed necessary. It was therefore agreed that semi-structured group interviews (dyads/triads) would be the most appropriate and effective method of data collection. The questions posed to participants (see Appendix A) built upon the findings of a pre-existing survey in order to gather a better understanding of the issues the survey had raised. These were the impact on individuals’ mood specifically, broader emotional health and well-being improvements derived from attendance, through to daily structure and information sharing.

To maximise uptake, the two group interviews were both held outside of working hours and scheduled to last no more than one hour and, (due to the geographically dispersed nature of participants and researchers) were conducted online. They were conducted on Zoom© version 5.17.11 [37], as participants were all familiar with the platform and it has been successfully employed in other studies both during and post-COVID [38]. To improve continuity and understanding, there was a short discussion held with participants about the format and structure of the interviews and to allow any pertinent queries to be dealt with prior to beginning. When this had been undertaken, the researchers (DB and CC) took turns to ask and direct the questions to ensure all participants were give a voice. Once completed, the online recording was stored securely on a dedicated research server at Sheffield Hallam University. Transcriptions of the recordings were subsequently produced using Microsoft Word before being reviewed by both researchers to ensure they were a true verbatim account of both discussions.

### 2.4. Data Analysis

Following transcription, the contents of each session were thematically analysed by way of Braun and Clarke’s [39] six-step thematic analysis method, as detailed below.

1. The facilitators familiarised themselves with the raw data from the interviews.
2. Initial codes were identified from the data.
3. Initial themes were generated from within the data.
4. The themes identified were reviewed against the specific aims of the evaluation.
5. The content and names of themes were reviewed for homogeneity and compatibility.
6. Suitable participant quotes were identified to aid accurate and illustrative write-up of each theme in this report [40].

N.B. Through stages four and five, to strengthen the analysis, the transcripts were initially analysed independently by the two facilitators (DB and CC). Each set of themes was then compared, discussed, and adjusted (by DB, CC, and JP) to create a final consensus.

### 2.5. Ethics

Sheffield Hallam University granted ethics approval for the study (ER:59716880). Established support group attendees were invited to participate. All who responded were emailed a participant information sheet and required to give written consent through an online Qualtrics© form [41]. Additionally, informed consent was confirmed verbally from all participants at the start of their group interview, following a thorough explanation of the evaluation’s purpose and an opportunity for questions. Participants were assured of the voluntary nature of their involvement and briefed on the importance of confidentiality regarding both their own data and others’ views shared during the meeting, as identified within data protection frameworks. They were also informed of their right to withdraw at any point during the session, with guidance provided on how to seek emotional support if necessary. Furthermore, participants were informed of the timeframe within which they could withdraw their data, up to two weeks following data collection, after which removal would be impractical due to transcription and analysis processes having commenced.

Both facilitators of the interviews (DB and CC) were lecturers in nursing at Sheffield Hallam University holding active registration with the Nursing and Midwifery Council. One of the facilitators (CC) was a mental health nurse, meaning they were confident to provide support/direction to other services post-session should any issues or distress arise as a result of the questions asked.

## 3. Results

In total, there were four participants who took up the invitation to attend the group interviews (one female and three male). The initial dyadic interview involved one female and one male, whilst the subsequent triadic interview comprised three males. One participant attended both sessions, and all participants were previous attendees of the PeerTalk support group meetings.

Following Braun and Clarke’s [39] thematic analysis method, a total of five key themes emerged, with a further two lesser themes considered areas for development: ‘talking/listening’; ‘socialising’; ‘contrast with other services’; ‘personal benefits’; ‘structure and accessibility’; ‘wider benefits’; and ‘areas for improvement/development’. These are represented pictorially as a coding tree in Appendix B, as well as being described in more depth below.

### 3.1. Talking/Listening

This theme considered the benefits of attending for all participants who engaged with the support groups. Attendees were able to consider and contextualise the benefits of sharing their experiences of common mental health problems in an empathetic and supportive manner. Participants felt that they could, at times, identify a tacit sense of solidarity with others: that they were not alone in the difficulties that they experienced and that this was beneficial to them and indeed others:

“…some people come and they don’t say a word. They’ve no need to speak; they just want to sit there and listen”.

(Participant 2)

“… it was quite relieving. Just being able to have a little space just to talk”.

(Participant 3)

Participants also identified an ability to offer a different perspective on peers’ experiences of addressing the everyday impact of their mental health difficulties. This is clearly in keeping with a peer support environment. It also confirms PeerTalk as a positive environment for addressing the impact that mental health difficulties can have on their everyday lives:

“I think these meetings allow people one, to voice what they think; and two, to reassure other people…”.

(Participant 2)

“It is an opportunity to talk about things that make me emotionally upset”.

(Participant 5)

“…helps you regulate it a bit, because once you’ve said it, it’s out in the world and it’s just like a problem halved”.

(Participant 3)

Finally, it was identified that individuals benefitted from the exchange of their personal experiences and that this was an environment that is conducive to this process:

“To have a genuine exchange of peer experiences…”.

(Participant 2)

### 3.2. Socialising

This theme considered how social networks play an important part of this process. The nature of a peer support system or support group is that individuals feel welcomed and are able to engage in a process of socialising. There was a clear demand for this type of service within each locality. Individuals identified that social contact with other people experiencing similar mental health difficulties was a significant value to them. Social isolation and a lack of social networks were regularly identified as being problematic for the individuals who experienced a mental health difficulty:

“…meeting people from all walks of life, you would never ever meet in any other circumstance”.

(Participant 2)

The groups highlighted the diverse range of support group attendees (in relation to life experiences, age, and gender). Individuals each brought with them different life experiences, backgrounds, and advice, which they imparted to others who were in attendance:

“The age group could be from 19 to, I think the group I go to, the oldest is 65”.

(Participant 2)

“…there’s a diverse range of people come to these meetings…”.

(Participant 2)

Individuals were treated as just that, individuals, whatever the difficulties they experienced in life. This environment was clearly non-judgemental in its approach and acceptance of others:

“…in PeerTalk, we’re totally neurodivergent when we totally just normally accept that, you know, that they’re, you know, the 16 others, as there often is, and in our group, there are 16 different versions of what’s going on…”.

(Participant 5)

“I get the perspective of these different people, you know, the women, as opposed to men, the young ones, the older ones, the students as opposed to the bricklayers, the whatever, and they all had this different perspective”.

(Participant 5)

Participants of both sessions conveyed a strong sense of camaraderie and solidarity—clearly supporting each other. This painted a picture of a friendly and warming environment that promoted the growth and spirit of togetherness:

“…get to know them as you come more regularly and I have made friends from it as well. It’s a friendly atmosphere”.

(Participant 2)

It was acknowledged that for newcomers to the group, it could seem daunting to enter this type of situation. However, it was argued that as the group became a focal point of peoples’ weekly routines, these concerns soon dissipated and became replaced by a feeling of less loneliness in life:

“Everyone’s nervous the first time—I was, but once you get to know how friendly it is, your loneliness dissipates because you’ve got this to look forward to every week”.

(Participant 6)

“And once you do it once [talk], it kind of breaks the barrier and then it’s easier and easier and easier”.

(Participant 3)

### 3.3. Contrast with Other Services

This theme considered how conversations and trails of discussion compared PeerTalk to other services, including those statutory services typically provided by psychiatric nurses and other mental healthcare professionals. PeerTalk is an independent charity, whereas many mental health services within the United Kingdom are predominantly provided by the National Health Service. Some participants talked about their experience of engaging with such services in comparison to PeerTalk. Many discussions focused on the length of time needed to wait to see, for example, a mental health professional and subsequent time for service provision:

“…waiting times can be hellishly long because of the demand…”.

(Participant 2)

“…when you’re waiting for something on a list, you think it’s never going to happen”.

(Participant 2)

“I’ve been to other groups where they give you two minutes or they give you a certain question to respond to. That’s not how mental health works. PeerTalk’s different”.

(Participant 6)

“It’s vital while people are waiting desperately to be treated by NHS services”.

(Participant 6)

Individuals also felt that the benefit gained from their attendance at a PeerTalk support group could be both significant and almost instantaneous:

“I think when I first went, I was struggling in between my mental health teams and stuff and I was just like in limbo”.

(Participant 3)

“I get far more from PeerTalk groups than I ever did with CBT”.

(Participant 2)

The timing of PeerTalk sessions was also of significance, with participants finding advantages over traditional mental health services that tended to be ‘9 to 5’ services:

“…there’s not enough help with mental health services out of hours”.

(Participant 2)

“If something happens with my mental health team, I can at least fall back on that [referring to PeerTalk] and I won’t have nothing”.

(Participant 3)

Participants acknowledged that Peer support groups were not traditional therapy and were deliberately non-medical in their approach. They perceived a clear distinction between service provision from the NHS and the service offered by PeerTalk:

“When I first came to a peer group, they were very careful to explain to me it is not a therapy”.

(Participant 5)

“Ultimately we’re not medical professionals; we’re not there to diagnose issues”.

(Participant 2)

### 3.4. Personal Benefits

This theme considers that participants in both group interviews identified a number of personal benefits that they were able to take away from their sessions. These benefits focused on building self-confidence and boosting self-esteem and encouraged individuals to engage in a personal journey of self-discovery:

“It’s helped me to quite significantly mature my self-perception and I’ve found some new insights”.

(Participant 2)

“I’ve been very grateful for my people, for giving me vocabulary and some labels to discuss those things”.

(Participant 5)

“It helps me to help others, to understand their feelings”.

(Participant 6)

“I experienced that feeling of being helpful and knowledgeable and important, and not just old and ill, and that’s greatly helped my self-confidence”.

(Participant 5)

Individuals voiced the notion of instilling hope. It was clear that participants had hope instilled by their attendance at a PeerTalk session, and that this was of huge significance to them:

“…because I’m involved in a group of people who’ve got future plans, they’re keeping up to date with themselves. I’m in the future and in the future, I have hope”.

(Participant 5)

Participants discussed the opportunity to build connections with others around them, similar to the notion of socialising; however, this extended beyond simply socialising with others. Individuals clearly derived significant benefit from attending the session:

“You’re building these connections with people helping them; you’re also helping yourself”.

(Participant 3)

Similarly, participants gained personal satisfaction and a boost to their own self-esteem from attending. Indeed, similarly, it may be suggested that PeerTalk evokes a sense within participants to actively engage with others who may be experiencing distress or difficulty:

“The biggest benefit for me is trying to help other people”.

(Participant 2)

### 3.5. Structure and Accessibility

This theme considered how discussion within the interviews highlighted that support groups helped to create their weekly routine, which was important to them and helped provide a structure to their week. Their PeerTalk groups became a constant feature in their lives, which again provided participants with a regular point of contact:

“…so same time every week, same place. I know I can always fall back on it”.

(Participant 3)

“In terms of structure, PeerTalk meetings give me a reason to have structure in the rest of my week. So I can talk about it”.

(Participant 6)

“But it’s so good to know my pattern is set one event after another. Yeah, 7:30 on a Thursday is very important”.

(Participant 2)

“I relish going to these groups ever single week and I go every single week without fail”.

(Participant 2)

### 3.6. Wider Benefits

This theme considered that it is important to acknowledge that there were several other disparate benefits that participants associated with their attendance at PeerTalk support group sessions. Whilst these did not particularly align with any of the previously identified themes, it was deemed important that they were not overlooked.

Participants consistently identified that humour within the groups was important to them:

“…we laugh quite a lot”.

(Participant 3)

But so was the generalised feeling of warmth, support, and genuine care for each other within the spirit of peer support:

“…you sometimes feel that you’re the only one, when actually there’s lots of people out there who need similar support”.

(Participant 2)

Participants also acknowledged the individuality of each attending member. Their problems were unique to them, and it was felt that these were never trivialised by other members. Additionally, participants also highlighted a sense of solidarity with each other:

“…you can see a different perspective that someone in that situation can’t see”.

(Participant 3)

“…if it’s not trivial to the individual, it’s so important they’re made aware of that”.

(Participant 2)

## 4. Discussion

From the two group interviews, five main themes were identified along with two lesser themes. These were: ‘talking/listening’; ‘socialising’; ‘contrast with other services’; ‘personal benefits’; ‘structure and accessibility’; ‘wider benefits’; and ‘areas for improvement/development’.

COVID-19 has had a lasting impact on the UK population. While the pandemic has subsided, some effects have continued even as the nation has returned to a degree of normality. What could be argued is that the isolation and loneliness evident prior to the pandemic has been exacerbated. The National Confidential Inquiry into Suicide and Safety in Mental Health [42] considers the number of deaths classed as suicide between the years of 2011 and 2021 as being 69,420, and interestingly highlights a decrease in suicide figures during the initial phases of the COVID-19 pandemic. One socio-demographic characteristic identified in the study features individuals living alone and being socially isolated. Peer support groups provide essential contact between participants (theme 2: socialising) with an exchange of information, support, and regular human connection [43].

A philosophy that peer support groups are recovery-focused is essential for the successful implementation of a support system for individuals who are experiencing mental health difficulties [44]. Individuals participating in the group interviews identified issues such as routine and structure. Having a daily routine to adhere to is important for any individual (with or without a mental health difficulty). One study [45] found that during the pandemic, there was a significant increase in individuals’ depressive symptoms due to their inability to fulfil their usual routines. The very nature of the support groups (being weekly on set days and at set times) provides this structure, which can maintain and possibly even improve symptoms of depression. In addition, there are benefits of forming and maintaining connections with others, primarily concerning features of their mental health difficulties (linking in with the themes of structure and accessibility, as well as personal gain). PeerTalk prides itself organisationally on being a ‘support’-focused group: many people attend due to where they are in their own lives—and recovery may well occur due to this.

Vickery [46] highlighted the benefits that peer support groups can have on individuals experiencing mental health difficulties, particularly men, and how they can provide not only an alternative source of personal support but also reciprocal support to others. Similarly, Satinsky et al. [47] discuss the importance and value of the individual within a two-way peer support process; and how individuals with well identified self-efficacy can feel empowered within their own recovery. Poor self-esteem is widely acknowledged to be associated with mental health difficulties [48,49]. Therefore, strengthened self-esteem is likely to provide positive outcomes. Through the group interviews and their qualitative analysis, it became clear that attendees and participants of PeerTalk groups achieve a sense of both belonging and empowerment, and that there is a clear exchange of support, shared and lived experiences, between all attendees (again identified within the themes of personal benefits, socialising, and talking and listening). This chimes with the concept of hope. Haim-Livetsky et al. [50] suggest that it is this very connectedness and belonging that provides hope. When considered within the symptom profile of depression, this is vital, as individuals who may be struggling with their mood often feel hopeless.

It is important to highlight that the peer support groups facilitated by PeerTalk are not a traditional treatment pathway and that the service is independent from the NHS. However, comparisons were drawn by participants to statutory mental health support, which identified shorter/non-existent waiting times. This is in stark contrast to NHS statistics released in 2024, showing that 89.3% of referrals to NHS talking therapies could take up to six weeks to be seen, and sometimes longer to start treatment [51]. This was highlighted within our theme of ‘contrast with other services’.

Whether as an adjunct or a complete alternative to statutory services, participants clearly derive benefit from attending PeerTalk sessions, with one attendee stating that they knew that this was not a form of counselling, but that the power of the group could not be ignored. Kelly et al. [52] investigated the benefits of attending a mutual self-help organisation within the United States and found outcomes were up to 20% better for individuals attending a support group versus cognitive–behavioural therapy alone.

### 4.1. Limitations of the Study

An obvious limitation to this study was the modest number of (primarily male) participants who engaged in each of the group interviews. However, this type of qualitative method does not require large numbers of participants, instead relying on the collection of rich data [53]. Furthermore, having had time to reflect, one of the initial dyad attended the subsequent session, as they were keen to offer some additional insights. Although methodologically challenging, this was accommodated, as it was deemed to be a reasonable adjustment in keeping with the Equality Act of 2010 [54]. Additionally, the use of online technology may have influenced uptake and hence the representativeness of our sample. However, since the COVID-19 pandemic, Zoom has become almost ubiquitous and is now commonly utilised in research studies [38]. Finally, participants were asked to self-identify as ‘regular’ attendees with sufficient experience to offer meaningful opinions rather than using a more standardised/quantitative criterion. Whilst these types of real-world issues inevitably limit the transferability of these results to other organisations, we would argue that the in-depth findings remain noteworthy, as they clearly highlight the way peer support services can helpfully complement the interventions typically provided by nurses and other healthcare professionals.

### 4.2. Recommendations for PeerTalk

Despite the limitations outlined above, it is reasonable to state that support group attendees derived many benefits from their attendance and that their overall experience was positive. That said, as with any type of service evaluation, it is important to create a well-rounded, objective overview of the service in question. In this regard, the main point of constructive criticism was concerning the numbers of participants attending each PeerTalk session. Participants explained that at times, attendance could be low and an increase in the number of people would increase the effectiveness of the group and its dynamics:

“…in a sense, the numbers need to be ramped up, to have a genuine exchange of peer experiences”.

(Participant 2)

“…the biggest negative is the lack of punters”.

(Participant 2)

### 4.3. Recommendations for Practice

Healthcare professionals have a unique position wherein they undertake many different concurrent roles when providing support to people with mental health conditions [55]. Directing to community self-help/peer support (or social prescribing) is just one of the activities they frequently undertake [56]. Nurses and other healthcare professionals should therefore familiarise themselves with the available local and national services, and through small-scale studies such as this, appreciate the benefits they can offer over and above their own highly pressured NHS provision.

## 5. Conclusions

As far back as 2013, Walker and Bryant [57] undertook a meta-synthesis of peer support services that was generally supportive of the concept; however, from our analysis, it is clear that these benefits are varied, context-specific, and idiosyncratic. This variability means small-scale evaluations such as ours have merit locally and further afield. In a post-pandemic society, this study has helpfully articulated the continued need for peer support services and highlighted the benefits gained by participants who attend. These include an effective way to combat loneliness and isolation, instilling hope in people for whom hope has been robbed by mental illness, a speedy alternative/adjunct to statutory services, and promoting recovery and self-efficacy. Many of the participants of the peer support groups we interviewed gain value not only from sharing their own experiences but also from helping others. It is perhaps within this symbiotic relationship that the true value of peer support lies. In light of their continued relevance and value, nurses and other healthcare professionals have a moral and professional responsibility to educate themselves about peer support and proactively direct service users to local groups wherever possible.

## Figures and Tables

**Figure A1:**
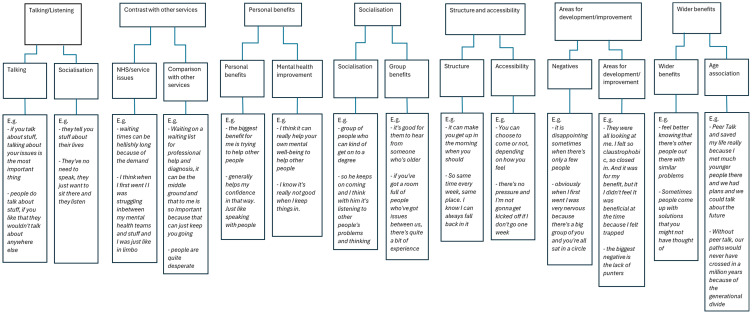
Coding tree (including number of codes per theme and example quotes).

## Data Availability

The data presented in this study are available on request from the corresponding author to maintain participant confidentiality.

## Public Involvement Statement

Patient partners were originally involved via a pre-existing survey in order to gather a better understanding of the issues. The information gathered from this survey was used to define the group interview questions. The report was disseminated to the patient partners once completed for feedback prior to finalisation.

## Guidelines and Standards Statement

This manuscript was drafted following Allison et al.’s “Consolidated criteria for reporting qualitative research (COREQ): a 32-item checklist for interviews and focus groups” (*International Journal for Quality in Health Care*, Volume 19, Issue 6, December 2007, Pages 349–357, https://doi.org/10.1093/intqhc/mzm042) [58].

## Appendix A

**Table A1:** Small-group interview questions.

Question No	Question
1	What is it about the sessions that provides benefit to you?
2	How have the PeerTalk sessions helped you relieve the pressure of your emotions?
3	Can you tell me how the PeerTalk sessions have helped with your confidence?
4	How have you been able to help support other group members?
5	How do the sessions help lift your mood?
6	How have the PeerTalk sessions helped you with loneliness?
7	In what way does the PeerTalk sessions provide structure to your life?
8	How has the PeerTalk sessions helped you understand your own feelings?
9	In what way do the sessions provide you with a new perspective on life?
10	What other support have you found through the PeerTalk sessions (e.g., other groups, services)
